# Effects of feeding α-amylase-expressed corn silage and grain on performance, enteric methane production, and carcass characteristics in beef steers

**DOI:** 10.1093/tas/txae080

**Published:** 2024-05-07

**Authors:** Lucas R Rebelo, Kirsten L Clark, Alejandro E Relling, Chanhee Lee

**Affiliations:** Department of Animal Sciences, The Ohio State University, Wooster OH 44691, USA; Department of Animal Sciences, The Ohio State University, Wooster OH 44691, USA; Department of Animal Sciences, The Ohio State University, Wooster OH 44691, USA; Department of Animal Sciences, The Ohio State University, Wooster OH 44691, USA

**Keywords:** beef cattle, Enogen corn as silage, Enogen corn as grain, processing of corn

## Abstract

An experiment was conducted to evaluate the effects of feeding Enogen feed corn (**EFC**) silage or EFC grain with different grain processing (dry-rolled corn vs. whole-shelled corn) in feedlot cattle diets. Total 68 Angus cross-bred steers were blocked by body weight and the treatments (diets) were randomly assigned to steers in each block: a basal diet with isoline corn silage and isoline dry-rolled corn grain (**IIR**); the basal diet with EFC silage and isoline dry-rolled corn grain (**EIR**); the basal diet with EFC silage and EFC dry-rolled grain (**EER**); and the basal diet with EFC silage and EFC whole-shelled grain (**EEW**). Isoline refers to the isogenic counterpart of Enogen corn silage or grain. Steers received the assigned treatment over 32 wk of the entire experiment (backgrounding and finishing) until harvested. Part of the steers (eight blocks) in each treatment were used to measure CH_4_ production (g/d) using the GreenFeed and CH_4_ production per unit of DMI. All data were analyzed using a mixed procedure of SAS in a randomized complete block design, considering diet as a fixed effect and block as a random effect. Steers fed the EIR diet increased (*P* = 0.03) DMI compared to IIR during the backgrounding phase. However, feeding EFC silage or grain did not affect body weight, average daily gain, and feed efficiency during backgrounding and finishing phases. Feeding EEW decreased (*P* ≤ 0.05) body weight, average daily gain, feed efficiency, and tended to decrease (*P* = 0.06) hot carcass weight compared to EER during the finishing phase. Methane production per unit of DMI decreased (*P* = 0.02) for steers fed EIR compared with steers fed IIR only during the backgrounding phase. Feeding EFC grain had no effect on CH_4_ production (g/d) in both phases. In conclusion, feeding EFC silage or grain did not improve the performance of beef steers during the backgrounding and finishing phases in the current experiment condition. Methane production per unit of DMI was reduced for steers fed EFC silage compared with isoline corn silage only during the backgrounding phase.

## Teaser

Feeding corn silage and corn grain with a trait that expresses high α-amylase may have potential to increase dietary starch digestibility and may improve the growth and performance of beef cattle. In the current study, we investigated feeding corn silage and grain with high α-amylase on performance and enteric methane production of beef steers.

## Introduction

A corn trait that expresses high α-amylase in corn kernel has been developed, Enogen corn for feed (EFC), which has potential to increase digestibility of starch and provide more rumen-available energy when fed to cattle compared with conventional corn. In an in vitro study, dry-rolled EFC showed greater degradation by rumen microbes compared with conventional dry-rolled corn (Horton and Drouillard, 2018). In an in vivo study, increases in feed efficiency and marbling scores were observed in finishing beef cattle fed dry-rolled EFC compared with those fed conventional dry-rolled corn (Jolly-Breithaupt et al., 2019). Growing beef calves fed EFC grain (dry-rolled or whole-shelled) had greater feed efficiency and tended to have greater ADG compared with those fed conventional corn grain in an experiment by Johnson et al. (2018). In another experiment by the same group (Johnson et al., 2019); however, no effect of feeding dry-rolled EFC (vs. conventional dry-rolled corn) were observed on growth or feed efficiency in growing calves, while EFC silage improved growth and feed efficiency. Volk et al. (2021) analyzed data from multiple studies (a total of 200 pen observations from seven experiments) to examine effects of dry-rolled EFC with varying proportions of distiller grains in diets of finishing beef cattle. In that study, cattle fed dry-rolled EFC increased feed efficiency, but the response to feeding dry-rolled EFC decreased as the proportion of distiller grains increased in the diet. The authors (Volk et al., 2021) concluded that feeding EFC as dry-rolled corn generally improved feed efficiency with a diet containing distiller grains, but the response to EFC was dependent on the inclusion rate of distillers grains. More studies are needed to understand inconsistent responses to feeding EFC.

In many small- and medium farms in Midwest US, it is popular to feed whole-shelled corn instead of processed corn, e.g., dry-rolled corn, to beef cattle simply because of low cost and lack of infrastructure to process. However, feeding whole-shelled corn may compromise the performance of beef cattle (e.g., growth and carcass; Freitas et al., 2020, 2021). Corn grain with high α-amylase activity, such as EFC, has potential to be fed as whole-shelled corn without compromising performance. Glaser et al. (2022) conducted two growth performance experiments with growing steers examining EFC silage and grains. The first experiment examined the effects of sources of corn grain (EFC vs. conventional) with different processing (dry-rolled vs. whole-shelled). As a result, EFC grain vs. conventional corn grain had positive responses of growth performance, but no processing effect was observed (Glaser et al., 2022). There was no interaction between the source and processing so the authors concluded that EFC whole-shelled grain was as good as conventional corn grain dry-rolled. The second experiment (Glaser et al., 2022) examined the effect of feeding EFC silage and grain (dry-rolled), and greater growth performance of steers fed EFC silage was observed, but no effect of feeding EFC grain was observed. Those experiments evaluated EFC on performance of only growing steers.

The objectives of the current experiment were to determine the effects of feeding EFC silage or EFC grain on performance and enteric methane production in beef steers during the growing and finishing phases. In addition, the effects of feeding whole-shelled EFC vs. dry-rolled EFC were determined. We hypothesized that feeding EFC silage or grain will improve feed efficiency potentially by improving starch digestibility, and this would lead to greater performance and less enteric methane production in growing and finishing beef steers. In addition, it was hypothesized that the performance of steers would be affected by the processing of EFC corn.

## Materials and Methods

The experiment was performed at Beef Research Center at the College of Food, Agricultural, and Environmental Sciences, Wooster campus, The Ohio State University. All procedures involving animals and their care were approved by the Institutional Animal Care and Use Committee of The Ohio State University (2021A00000038).

## Experiment Design and Treatments

A total of 68 Angus beef steers after weaning (mean ± SD; 250 ± 36.5 kg of BW; 7.1 ± 0.6 mo age) arrived at the research facility and were assigned to individual stalls. All animals used in this study were raised and weaned at The Ohio State University beef farm branches. During the first 2 wk upon arrival, all animals were weighed, ear-tagged, and received Inforce 3 (Zoetis, Parsippany, NJ) for the prevention of respiratory diseases and Vetmetric pour-on (MWI veterinary supply; Northern Ireland) for the protection of parasitic infection at the beef research facility. All steers received a standard growing diet during the first 2 wk upon arrival as an adaptation to the stalls and environment. At the end of the first 2 wk, all steers were weighed on 2 consecutive days and blocked by BW, and animals in each block were randomly assigned to one of four treatments (e.g., 17 blocks, four animals per block). All animals remained in their assigned treatments from backgrounding through finishing phase. The backgrounding phase lasted for 9 wk during which the following treatment diets were fed to steers: a basal diet with isoline corn silage and isoline dry-rolled corn grain **(IIR**); the basal diet with EFC silage and isoline dry-rolled corn grain (**EIR**); the basal diet with EFC silage and EFC dry-rolled corn grain (**EER**); and the basal diet with EFC silage and EFC whole-shelled corn (**EEW**; Table 1). Isoline refers to the isogenic counterpart of Enogen corn silage or grain. After 9 wk of the growing phase, the basal diet was switched gradually to a typical finishing diet over 3 wk (Table 1). During the transitioning phase, the proportion of concentrate increased (43.4% to 87.8%) and that of forage decreased gradually (56.6% to 12.2% on a DM basis). The finishing phase followed the transition phase and lasted for 20 wk. Therefore, the entire experiment lasted for 32 wk. Enogen and isoline corn were planted, harvested, and processed for silage and grain similarly at The Ohio State University (Wooster, OH). Corn plants harvested were chopped (theoretical length of cut, 17 mm), and ensiled in silo bags (2.7 m in diameter with about 6.3 m^3^ of filling per running meter) in September 2020. Both corn hybrids were allowed to ferment 40 d before opening. Corn grain was harvested in November 2020 and stored in grain storage (gravity wagons) until processed (dry-rolled or whole-shelled) before feeding. During the entire experiment, all steers were fed the diets for ad libitum intake (target of 2% refusal) with free access to water.

**Table 1.** Dietary ingredients and chemical composition

	Backgrounding diets^*^				Finishing diets^*^			
Ingredients (DM, %)	IIR	EIR	EER	EEW	IIR	EIR	EER	EEW
Isoline corn silage	56.6	0.0	0.0	0.0	12.2	0.0	0.0	0.0
EFC silage^†^	0.0	56.6	56.6	56.6	0.0	12.2	12.2	12.2
Isoline corn, dry-rolled grain	18.3	18.3	0.0	0.0	66.3	66.3	0.0	0.0
EFC, dry-rolled grain ^†^	0.0	0.0	18.3	0.0	0.0	0.0	66.3	0.0
EFC, whole-shelled grain^†^	0.0	0.0	0.0	18.3	0.0	0.0	0.0	66.3
Soybean meal	10.0	10.0	10.0	10.0	8.4	8.4	8.4	8.4
DGGS^‡^	12.5	12.5	12.5	12.5	8.9	8.9	8.9	8.9
Minerals/vitamins^∥^	1.85	1.85	1.85	1.85	3.47	3.47	3.47	3.47
Trace mineral mix^$^	0.75	0.75	0.75	0.75	0.68	0.68	0.68	0.68
Chemical composition (% of DM)								
OM	93.0	93.1	93.1	93.1	94.7	94.7	94.4	94.4
CP	14.5	14.2	14.2	14.2	13.0	12.9	12.4	12.4
NDF	31.1	29.4	29.3	29.4	14.8	14.8	14.9	14.9
Starch	32.6	34.5	34.4	34.4	51.9	51.8	53.3	54.3
Ca	0.99	0.99	0.99	0.99	1.03	1.03	1.04	1.03
P	0.41	0.40	0.40	0.40	0.36	0.36	0.37	0.37

^*^IIR, Isoline corn silage and Isoline dry-rolled corn grain; EIR, Enogen corn silage and Isoline dry-rolled corn grain; EER, Enogen corn silage and Enogen dry-rolled corn grain; EEW, Enogen corn silage and Enogen whole-shelled corn grain. The experimental diets were fed for 9 wk of the backgrounding and 20 wk of the finishing phase.

^†^EFC, Enogen feed corn.

^‡^Dried corn distillers’ grains with solubles (Poet Nutrition Inc.).

^∥^The mix contained (DM basis) 0.54% vitamin A, 0.01% vitamin D, 4.73% of vitamin E, 0.07% of copper sulfate, 33.8% of calcium phosphate, and 60.8% of limestone for the backgrounding and contained 0.36% vitamin A, 0.003% vitamin D, 2.72% of vitamin E, 0.05% of copper sulfate, 6.1% of calcium phosphate, and 90.8% of limestone for the finishing.

^$^The premix contained (as-is basis) 39.3% sodium, 53.2% chloride, 70 mg/kg cobalt, 400 mg/kg copper, 70 mg/kg iodine, 1,750 mg/kg iron, 2,800 mg/kg manganese, and 3,500 mg/kg zinc (Morton Salt Inc.).

## Sampling and Measurements

Diets were prepared every morning and offered as TMR to individual steers at 0900 hours once daily. Feed refusals were weighed daily to measure daily intake. Individual feed ingredients (i.e., forages, corn grains, and concentrates) were collected monthly for analyses of chemical composition. Samples were submitted to Rock River Laboratory (Watertown, WI; https://rockriverlab.com) for standard chemical analyses (Table 1 and 2), i.e., DM (105 °C at 3 h), OM (method 942.05, AOAC International, 2000), NDF with amylase and sodium sulfite (Goering and Van Soest, 1970) using Ankom200 Fiber Analyzer (Ankom Technology Corp), starch (Hall, 2009), and minerals (ICP-OES after acid digestion). Samples of feed refusals were collected weekly and composited by animal and month for DM analysis to calculate daily DMI.

**Table 2.** Chemical composition of corn silages and corn grains (% of DM if not specified)

Items	Isoline corn silage	EFC corn silage	Isoline corn, dry-rolled grain	EFC, dry-rolled grain	EFC, whole-shelled grain
DM, % of as-is	39.2	42.4	85.1	83.9	84.1
OM	96.2	96.0	98.6	98.4	98.4
CP	7.8	7.5	8.5	8.0	8.0
NDF	36.4	36.3	7.9	8.0	8.1
Starch	36.6	36.4	68.1	69.3	70.7
Ca	0.14	0.14	0.03	0.04	0.03
P	0.13	0.18	0.26	0.30	0.30

EFC, Enogen feed corn.

Body weights were measured on 2 consecutive days every 3 wk during the entire experiment. Growth, daily gain, and feed efficiency were calculated for individual animals using BW and feed intake. All steers were finished at a common days-on-feed, shipped to a local commercial abattoir for slaughter. Individual animals were evaluated for carcass characteristics and values according to the USDA standard procedure by a USDA grader.

Eight blocks of steers were randomly selected (*n* = 8 animals per treatment) and used to determine enteric CH_4_ and CO_2_ production. Before measurements, individual steers were trained to use the GreenFeed (C-Lock Inc., Rapid City, SD) with bait feeds. The measurement was conducted in week 8 during the backgrounding phase. Each animal received eight measurements over 3 d in equally spaced time points to represent every 3 h in a 24-h cycle (Hristov et al., 2015). For each measurement, animals were offered about 200 g of a bait feed (sweet calf starter texturized, Kalmbach Feeds; 23% CP, 3.5% crude fat, and 12% ADF on a DM basis) to allow for at least 5 min of breath collection. The same previously assigned blocks of steers were used to measure enteric CH_4_ and CO_2_ production in week 18 during the finishing phase.

## Statistical Analysis

Steers were the experimental unit (20 per treatment). Data of DMI, BW, ADG, and G:F were analyzed using the MIXED procedure of SAS version 9.4 (SAS Institute Inc., Cary, NC). The model included the fixed effects of dietary treatment, week (repeated variable), and the two-way interaction between fixed effects and the random effect of block. The covariance structure of AR(1) for the repeated measure was used according to the lowest Akaike information criterion. For carcass characteristics and enteric methane, week effect (repeated) was removed from the model. All denominator degrees of freedom were adjusted using the Kenward-Rogers option. Preplanned contrasts were made to examine the effects of EFC silage (IIR vs. EIR), EFC dry-rolled grain (EIR vs. EER), or processed EFC grain (EER vs. EEW). Statistical significance was set as *P* ≤ 0.05 and statistical trends were declared as 0.05 < *P* ≤ 0.10. All data are presented as least squares mean.

## Results

During the entire experiment, the week effect on DMI, BW, and ADG was significant (*P* < 0.05), but none of the variables had an interaction of treatment by week. During the backgrounding phase, feeding EFC silage or grain did not affect BW, ADG, and G:F (*P* ≥ 0.19; Table 3). Dry matter intake was not affected by treatments (*P* ≥ 0.20) except that steers fed EIR had greater DMI (7.89 vs. 7.41 kg/d; *P* = 0.03) compared with steers fed IIR. During the finishing phase, DMI was not affected by treatments (*P* ≥ 0.17). However, BW (518 vs. 500 kg; *P* = 0.04), ADG (1.49 vs. 1.33 kg/d; *P* = 0.05), and G:F (0.152 vs. 0.141 kg/d; *P* = 0.03) were greater for steers fed EER compared with EEW. No difference in those variables (*P* ≥ 0.16) was observed between steers fed EIR and EEW, though. Enogen corn silage did not affect (*P* ≥ 0.19) growth of steers during the backgrounding phase. Enogen corn silage and grain did not affect (*P* ≥ 0.33) final BW and hot carcass weight (HCW; Table 4). However, steers fed EER tended to have greater final BW (594 vs. 572 kg; *P* = 0.09) and HCW (371 vs. 357 kg; *P* = 0.06) compared with steers fed EEW. Neither yield grade nor quality grade were affected by treatments.

**Table 3.** Effects of feeding Enogen corn silage with Enogen dry-rolled or whole-shelled corn grain on dry matter intake, body weights, and average daily gains

	Diets^*^					*P*-values^†^		
Items	IIR	EIR	EER	EEW	SEM	Silage	Grain	Processing
Backgrounding (63 d)								
DMI, g/d	7.41	7.89	7.61	7.90	0.240	0.03	0.22	0.20
BW, kg	311	315	314	315	2.15	0.19	0.84	0.78
ADG,^‡^ kg/d	1.61	1.70	1.66	1.71	0.052	0.21	0.55	0.54
G:F,^∥^ kg/kg	0.219	0.218	0.218	0.218	0.0063	0.85	0.92	0.96
Finishing (140 d)								
DMI, kg/d	9.60	9.33	9.83	9.38	0.261	0.45	0.17	0.22
BW, kg	514	514	518	500	6.91	0.99	0.60	0.04
ADG,^‡^ kg/d	1.47	1.38	1.49	1.33	0.057	0.22	0.16	0.05
G:F,^∥^ kg/d	0.153	0.147	0.152	0.141	0.0049	0.26	0.33	0.03

^*^IIR, Isoline corn silage and Isoline dry-rolled corn grain; EIR, Enogen corn silage and Isoline dry-rolled corn grain; EER, Enogen corn silage and Enogen dry-rolled corn grain; EEW, Enogen corn silage and Enogen whole-shelled corn grain. The experimental diets were fed for 9 wk of the backgrounding and 20 wk of the finishing phase.

^†^Silage, IIR vs. EIR; Grain, EIR vs. EER; Processing, EER vs. EEW.

^‡^Average daily gain.

^∥^Gain:Feed.

**Table 4.** Effects of feeding Enogen corn silage with Enogen dry-rolled or whole-shelled corn grain on carcass characteristics

	Diets^*^					*P*-values^†^		
Items	IIR	EIR	EER	EEW	SEM	Silage	Grain	Processing
Final BW, kg	592	582	594	572	11.5	0.37	0.33	0.09
HCW^‡^	369	365	371	357	7.5	0.60	0.43	0.06
Dressing, %	62.2	62.7	62.5	62.1	0.41	0.36	0.75	0.48
YG^∥^	3.81	3.98	3.82	3.74	0.176	0.46	0.47	0.75
12th-rib fat, cm	1.83	1.90	1.82	1.74	0.104	0.66	0.64	0.61
LM area,^$^ cm	80.3	77.8	80.7	78.7	1.46	0.21	0.15	0.31
Marbling score	744	763	706	734	25.8	0.60	0.12	0.44
KPH,^¶^ %	2.03	2.14	2.08	2.19	0.074	0.23	0.52	0.23
QG^**^	6.88	6.80	6.31	6.73	0.302	0.84	0.25	0.32

^*^IIR, Isoline corn silage and Isoline dry-rolled corn grain; EIR, Enogen corn silage and Isoline dry-rolled corn grain; EER, Enogen corn silage and Enogen dry-rolled corn grain; EEW, Enogen corn silage and Enogen whole-shelled corn grain. The experimental diets were fed for 9 wk of the backgrounding and 20 wk of the finishing phase.

^†^Silage, IIR vs. EIR; Grain, EIR vs. EER; Processing, EER vs. EEW.

^‡^Hot carcass weight.

^∥^Yield grade.

^$^Longissimus muscle.

^¶^Kidney, pelvic, and heart fat.

^**^Quality grade.

During the enteric CH_4_ measurements in the backgrounding phase, DMI tended to be greater for steers fed EIR (8.32 vs. 7.40 kg/d; *P* = 0.06) compared with IIR during the enteric CH_4_ measurement (Table 5). In addition, steers fed EEW tended to increase DMI (8.58 vs. 7.76 kg/d; *P* = 0.09) compared with EER. Enteric CH_4_ production was not affected by feeding EFC silage and grain, but CH_4_ production per unit of DMI was lower (23.4 vs. 29.1 g/kg DMI; *P* = 0.02) for steers fed EIR compared with IIR. During the finishing phase, DMI was greater (9.37 vs. 8.11 kg/d; *P* = 0.04) for steers fed EER compared with EIR. However, CH_4_ and CH_4_ per unit of DMI were not affected (*P* ≥ 0.18) by feeding EFC silage or grain.

**Table 5.** Effects of feeding Enogen corn silage with Enogen dry-rolled or whole-shelled corn grain on DMI, enteric methane, and carbon dioxide emissions in week 8 during the backgrounding and in week 18 during the finishing phase

	Diets^*^					*P*-values^†^		
Items	IIR	EIR	EER	EEW	SEM	Silage	Grain	Processing
	Backgrounding							
DMI,^‡^ kg/d	7.40	8.32	7.76	8.58	0.441	0.06	0.23	0.09
CO_2_, g/d	7,560	7,703	7,500	7,700	324.1	0.72	0.61	0.62
CH_4_, g/d	207	188	202	204	13.2	0.28	0.43	0.88
CH_4_ g/kg DMI	29.1	23.4	25.9	24.3	2.09	0.02	0.28	0.48
	Finishing							
DMI,^‡^ kg/d	8.44	8.11	9.37	8.69	0.444	0.60	0.04	0.27
CO_2_, g/d	10,552	10,013	10.523	10,800	578.8	0.44	0.45	0.69
CH_4_, g/d	135	137	162	166	13.4	0.96	0.18	0.85
CH_4_ g/kg DMI	16.6	17.9	17.6	19.6	2.14	0.69	0.95	0.53

^*^IIR, Isoline corn silage and Isoline dry-rolled corn grain; EIR, Enogen corn silage and Isoline dry-rolled corn grain; EER, Enogen corn silage and Enogen dry-rolled corn grain; EEW, Enogen corn silage and Enogen whole-shelled corn grain. The experimental diets were fed for 9 wk of the backgrounding and 20 wk of the finishing phase.

^†^Silage, IIR vs. EIR; Grain, EIR vs. EER; Processing, EER vs. EEW.

^‡^Measured during CH_4_ measurement over 3 d.

## Discussion

Although statistical analyses were not conducted, dietary nutrient composition during the backgrounding and finishing phase did not differ among treatments due to similar chemical composition between isoline and EFC silage and between isoline and EFC grain. In our previous study with dairy cows (Rebelo et al., 2023), chemical composition of isoline vs. EFC for silage and grain were examined whereas the chemical compositions of corn silage or grain between isoline and EFC were not different. No difference in the chemical composition of diets with and without EFC silage or corn grain was reported in studies with growing cattle or non-ruminants (Ochonski et al., 2021; Williams et al., 2021b; Glaser et al., 2022). Cueva et al. (2021) examined the effects of feeding EFC silage in dairy cows where EFC silage had greater starch (3% units) and lower CP (0.5% unit) compared with isoline corn silage.

During the backgrounding phase, an increase in DMI for steers fed EIR compared with IIR agrees with our previous study (Rebelo et al., 2023) where dairy cows fed EFC silage increased DMI by 1.5 kg/d compared with isoline corn silage. In addition, a recent paper where growing steers were fed EFC silage reported a tendency for increased DMI in a growth performance experiment but a decrease in DMI for growing steers fed EFC silage in another experiment (Glaser et al., 2022). No difference in DMI was observed in a study by Krogstad and Bradford (2023) when dairy cows were fed EFC silage. The increase in DMI for EFC silage observed in the current study is difficult to explain. However, our previous study with dairy cows (Rebelo et al., 2023) observed greater alpha-amylase activity in EFC silage and cows fed EFC silage tended to increase microbial protein synthesis. Perhaps, the feed digestion and passage rate in the rumen were greater for steers fed the diet with EFC silage. This hypothesis seems to be, at least in part, supported by no difference in DMI during the finishing phase when the proportion of EFC silage in the diet was much smaller compared with that during the backgrounding phase (12.2% vs. 56.6% of dietary DM). In the experiments conducted by Glaser et al. (2022), a growth performance experiment reported decreases in fecal starch concentration and another digestibility study reported increases in DM and OM digestibility for steers fed EFC silage compared with conventional corn silage. However, a study by Rebelo et al. (2023) showed no effect of feeding EFC silage on rumen and total tract digestibility of DM and NDF for dairy cows fed EFC silage. No differences in DMI by feeding EFC dry-rolled (i.e., EIR vs. EER) agrees with previous studies with dairy cows (Rebelo et al., 2023), pigs (Williams et al., 2021b, 2021c), finishing beef steers (Jolly-Breithaupt et al., 2019), and growing steers (Glaser et al., 2022). A tendency for decreased DMI for feeding EFC grain was also reported previously (Johnson et al., 2019; Jolly-Breithaupt et al., 2019). The mixed responses of DMI to EFC silage or grain warrant more studies.

Although DMI increased for steers fed EFC silage during the backgrounding phase, it did not result in increases in BW, ADG, and feed efficiency. In addition, the performance was not affected by feeding either EFC silage or dry-rolled grain during the backgrounding and finishing phase. To the best of our knowledge, only one study that evaluated EFC silage in beef cattle is available (Glaser et al., 2022). In that experiment, EFC silage was fed to growing steers for 91 d increased ADG and tended to increase BW. However, because of increased DMI, feed efficiency (G:F) was not affected by EFC silage. When EFC silage was fed to dairy cows, Rebelo et al. (2023) observed increased milk yield likely due to increased DMI compared with isoline corn silage. Cueva et al. (2021) observed an increase in milk yield of cows fed EFC silage without change in DMI. However, Krogstad and Bradford (2023) did not find an increase in the production of lactating cows fed EFC silage. More studies with beef cattle fed EFC grain are available. Volk et al. (2021) observed increases in feed efficiency when EFC dry-rolled grain was fed to finishing beef steers, but the positive response was dependent upon the level of byproducts in the diet, i.e., distillers grains or Sweet Bran^®^ (Cargill Wet Milling), and feeding EFC high-moisture grain did not improve ADG and G:F when compared with conventional high-moisture corn grain. Similar results were observed in an experiment by Jolly-Breithaupt et al. (2019) where steers fed EFC dry-rolled grain increased feed efficiency only when the diet included Sweet Bran. Another experiment by Jolly-Breithaupt et al. (2019) showed an increase in ADG and G:F for finishing steers fed EFC dry-rolled.

According to the studies in the literature, responses to EFC silage and grain have been variable and could be dependent upon the composition of basal diets. In a study by Volk et al. (2021), diminishing responses to feeding EFC grain as proportion of distillers grains increased in the diet may suggest that high polyunsaturated fatty acids supply from distillers grains affected microbial activity or efficiency negatively and ruminal microbes were not able to efficiently utilize starch for energy. Positive responses in feed efficiency to feeding EFC grain when Sweet Bran was included in the basal diets (Jolly-Breithaupt et al., 2019; Volk et al., 2021) indicates that starch availability was not the only condition to improve beef cattle performance. The authors in those studies did not provide a potential mode of action for positive feed efficiency with feeding EFC grain when fed with Sweet Bran. In the current study, because DMI and performance were not affected by EFC silage and grain during the finishing phase, carcass characteristics were also not influenced by those as well. Although the digestive physiology is largely different, when EFC ground grain was fed to finishing pigs or sows, no positive effect on performance was observed (Williams et al., 2021a, 2021b).

Although not affected during the backgrounding phase, increases in BW, ADG, and G:F during the finishing phase and a tendency for increase in HCW for steers fed EER compared with steers fed EEW suggests that processing of corn grain is a factor affecting performance of finishing beef steers. It has been recognized that corn processing influences starch digestibility and feedlot performance of cattle (Galyean et al., 1979; Corona et al., 2005). Positive performance responses to feeding dry-rolled corn vs. whole-shelled corn have been reported (Zinn et al., 2011; Freitas et al., 2021) although studies with no effect on performance are also available (Freitas et al., 2020; Carvalho et al., 2021). There are limited studies available on effects of feeding EFC grain differently processed. Volk et al. (2021) examined effects of feeding EFC dry-rolled or EFC high-moisture to finishing cattle. Average daily gain and G:F increased with feeding EFC dry-rolled compared with conventional dry-rolled corn, but feeding EFC high-moisture had no effect on performance of finishing cattle when compared with conventional high-moisture corn grain. Glaser et al. (2022) studied effects of corn grain sources (conventional vs. EFC) with different processing (dry-rolled or whole-shelled) in growing steers. In that experiment, feeding EFC grains increased or tended to increase ADG and G:F compared with conventional corn grains regardless of grain processing. Steers fed dry-rolled corn grain tended to increase ADG regardless of corn sources (conventional and EFC) and no interaction between corn source and processing was observed. The authors concluded that EFC whole-shelled grain was as good as dry-rolled EFC or conventional corn grain in terms of performance in growing steers. However, decreasing the particle size of corn grain improved the growth performance of litters of sows fed the corn grain, but either a difference in those between conventional and EFC grain or interaction of corn source by particle size was not observed (Williams et al., 2021a). In the current study, EFC dry-rolled grain was superior to EFC whole-shelled grain in fattening performance during the finishing phase, but we did not determine effects of feeding EFC whole-shelled grain compared with isoline dry-rolled corn grain in the current study. However, a pairwise *t*-test between EIR and EEW resulted in no difference in DMI, BW, ADG, and G:F (data not shown), suggesting that steers fed EFC whole-shelled grain could have had performance that was as good as those fed isoline dry-rolled grain.

Methane production and CH_4_ production per unit of DMI were not affected by feeding EFC silage and grain during the finishing phase. Perhaps, a further decrease in CH_4_ by feeding EFC silage and grain was difficult to achieve because steers were fed a high grain diet and produced relatively low CH_4_ during the finishing phase. To decrease CH_4_ production by feeding EFC silage and grain, the quantity or rate of starch digestion in the rumen should have been greater to lower rumen pH and fiber digestibility compared with isoline sources. Although the quantity or rate of starch digestion increased with EFC grain (Glaser et al., 2022), the increase might not have been sufficient to decrease enteric CH_4_ production under a high-grain diet. Previously, no effect of corn processing (whole, cracked, or steam-flaked corn) on enteric CH_4_ production was reported (Petzel et al., 2021). Keomanivong et al. (2017) conducted an in vitro experiment with rumen fluid from steers fed a high-concentrate diet to examine effects of corn processing (coarse vs. fine dry-rolled corn). In that study, CH_4_ concentration in the headspace of the in vitro chamber was not affected. However, a study by Hales et al. (2012) observed decreases in enteric CH_4_ production for finishing steers fed steam-flaked corn vs. dry-rolled corn.

During the backgrounding phase, feeding EFC grain did not affect CH_4_ production, but feeding EFC silage decreased CH_4_ production per unit of DMI by 20% compared with isoline corn silage. This occurred because EIR had numerically lower CH_4_ production (g/d) despite greater DMI compared with IIR. The results agree with our previous study (Rebelo et al., 2023) where dietary proportions of forage and concentrate in the diet fed to dairy cows were similar to that in the current study. When dairy cows were fed either EFC silage or EFC silage and grain, steers fed EFC silage decreased CH_4_ production per unit of DMI by 15% compared with isoline corn silage, while feeding EFC grain had no effect on CH_4_ production. In that study, EFC silage decreased CH_4_ production per unit of DMI although ruminal starch digestion was not affected. However, because microbial protein synthesis increased for steers fed EFC silage in that study, the authors speculated that the ruminal starch digestion rate, not the daily quantity of digestion, might have been improved. Furthermore, because microbes are another [H^+^] sink in the rumen, the authors assumed that the decrease in CH_4_ production per unit of DMI for EFC silage was, at least in part, attributed to the increase in microbial protein synthesis. This may have occurred for steers-fed EFC silage in the current study as well during the backgrounding phase. However, no effect of feeding EFC grain on CH_4_ production in Rebelo et al. (2023) and the current study may suggest that alpha-amylase activity or effectiveness of alpha-amylase from corn grain in the rumen was not as good as that from corn silage.

## Conclusion

Feeding EFC silage and grain did not improve the performance of beef steers during the growing and finishing phases in the current experiment, although DMI increased for steers fed EFC silage during the backgrounding phase. However, feeding EFC whole-shelled grain decreased BW, ADG, and feed efficiency and tended to decrease HCW compared with feeding EFC dry-rolled grain. Although not tested in this study, we observed the potential that steers fed EFC whole-shelled grain could have had performance that was as good as those fed isoline dry-rolled corn grain, requiring further studies. Methane production per unit of DMI was not affected by feeding EFC silage or grain during the finishing phase. However, feeding EFC silage decreased CH_4_ production per unit of DMI compared with isoline corn silage during the backgrounding phase where the proportion of forage in the diet was relatively high, which confirmed the results of our previous study with dairy cows.
